# Editorial: The tumor microenvironment and malignant properties in primary liver cancer

**DOI:** 10.3389/fonc.2023.1264439

**Published:** 2023-08-21

**Authors:** Chen Qu

**Affiliations:** Department of Pathophysiology, School of Medicine, Jinan University, Guangzhou, China

**Keywords:** tumor microenvironment, liver cancer, hepatocellular carcinoma, immunotherapy, prognosis

Primary liver cancer, predominantly hepatocellular carcinoma (HCC) and intrahepatic cholangiocarcinoma (ICC), is a leading cause of cancer-related mortality worldwide, characterized by its insidious development and poor prognosis. Over the years, it’s become apparent that a deeper understanding of the tumor microenvironment (TME) is crucial in decoding the pathophysiology of primary liver cancer and developing innovative therapies (1).

The TME is a complex mosaic of cancer cells, fibroblasts, immune cells, blood vessels, and extracellular matrix. It plays an influential role in malignant progression (2). It’s not just an inert bystander, but an active contributor in shaping cancer’s behavior, including primary liver cancer. Among the vital players in the TME, Immune cells have shown significant impact and therapeutic implications (3). Myeloid-derived suppressor cells (MDSCs) and regulatory T cells (Tregs) significantly contribute to immunosuppression in HCC, impeding the body’s anti-tumor responses. The manipulation of the immune component of the TME, as exemplified by the success of immune checkpoint inhibitors, heralds a promising avenue for targeted therapies. Cancer-associated fibroblasts (CAFs) are another central element of the TME (4). Acting as master regulators, they influence other components of the TME through the secretion of growth factors, cytokines, and extracellular matrix proteins, fostering an environment conducive to tumor growth. Their role in promoting angiogenesis, immune evasion, and metastasis emphasizes the necessity for a comprehensive understanding of CAFs in devising therapeutic strategies against primary liver cancer.

Despite the advancements in our understanding of the TME, a critical challenge is the heterogeneity within and between tumors. The dynamic interplay of cellular and non-cellular elements in the TME creates a multifaceted network, variable between patients and even within the same tumor. This complexity necessitates the development of personalized strategies for TME targeting. The advent of single-cell technologies has revolutionized our capacity to deconvolute this heterogeneity. By dissecting the TME at an unprecedented resolution, these technologies could provide novel insights into the pathogenesis of primary liver cancer and help identify potential therapeutic targets.

While the importance of the TME in primary liver cancer is widely accepted, our knowledge remains limited, and existing therapies have yet to fully exploit this potential. Immune checkpoint inhibitors, though showing promise, have only benefited a subset of patients. This calls for a more holistic approach, integrating knowledge from genomics, immunology, and cell biology to delineate the TME’s intricacies in liver cancer. Additionally, there is a growing need for developing robust preclinical models that accurately recapitulate the liver TME. While organoids and patient-derived xenografts represent significant strides, they fail to fully capture the complexity of the *in vivo* environment. Efforts towards refining these models and developing more physiologically relevant ones could revolutionize our ability to predict therapeutic responses and test novel drugs. Moreover, clinical trials should be designed to specifically interrogate the TME’s impact on therapeutic efficacy, biomarker development and incorporation into trial designs could enable stratification of patients most likely to benefit from TME-targeted therapies.

In this Research Topic, several works discussed the association between tumor microenvironment and malignant properties in primary liver cancer. Wang et al. discussed the differences between radiofrequency ablation and microwave ablation in inducing the immune regulation of NK cells for HBV-associated primary hepatocellular carcinoma. Isowa et al. reported that the addition of alkalization therapy may improve the efficacy of standard therapies in HCC patients. Liu et al. reported that KIF5A might serve as a potential biomarker for predicting immunotherapy response and could be a potential target for HCC. In addition, Liu et al. reviewed the cellular crosstalk in HCC TME. The four papers that comprise this Research Topic may help to understand the association between tumor microenvironment and malignant properties in primary liver cancer.

In conclusion, the TME is a critical determinant of malignant progression in primary liver cancer, underscoring the need for a concentrated research effort and a shift towards TME-centric therapeutic strategies. Leveraging novel technologies, developing physiologically relevant preclinical models, and designing innovative clinical trials hold the key to unlocking the potential of TME-targeted therapies in improving the prognosis of liver cancer patients. We must seize this opportunity to turn our increasing understanding of the TME into tangible benefits for patients battling this devastating disease.

## Author contributions

CQ: Writing – original draft.
